# Therapeutic biomarkers in acute myeloid leukemia: functional and genomic approaches

**DOI:** 10.3389/fonc.2024.1275251

**Published:** 2024-02-12

**Authors:** Karanpreet Bhatia, Vedant Sandhu, Mei Hsuan Wong, Prasad Iyer, Shruti Bhatt

**Affiliations:** ^1^ Department of Pharmacy, National University of Singapore, Singapore, Singapore; ^2^ Children’s Blood and Cancer Centre, KK Women’s and Children’s Hospital, Singapore, Singapore; ^3^ Duke-National University of Singapore (NUS) Medical School, Singapore, Singapore

**Keywords:** precision medicine, functional genomics, functional precision medicine, AML, precision oncology

## Abstract

Acute myeloid leukemia (AML) is clinically and genetically a heterogeneous disease characterized by clonal expansion of abnormal hematopoietic progenitors. Genomic approaches to precision medicine have been implemented to direct targeted therapy for subgroups of AML patients, for instance, IDH inhibitors for *IDH1/2* mutated patients, and FLT3 inhibitors with *FLT3* mutated patients. While next generation sequencing for genetic mutations has improved treatment outcomes, only a fraction of AML patients benefit due to the low prevalence of actionable targets. In recent years, the adoption of newer functional technologies for quantitative phenotypic analysis and patient-derived avatar models has strengthened the potential for generalized functional precision medicine approach. However, functional approach requires robust standardization for multiple variables such as functional parameters, time of drug exposure and drug concentration for making *in vitro* predictions. In this review, we first summarize genomic and functional therapeutic biomarkers adopted for AML therapy, followed by challenges associated with these approaches, and finally, the future strategies to enhance the implementation of precision medicine.

## Introduction

Acute myeloid leukemia (AML) is a heterogenous and molecularly complex blood and bone marrow malignancy. A median age of diagnosis for AML patients is about sixty eight years (1). The prognosis in the elderly AML (>65 years) patients is worse compared to younger patients (<65 years) with a 5-year median survival of less than 20% (2, 3). There has been a significant progress in understanding the biology of AML including identification of translocations leading to aberrant fusion proteins such as t(8:21) (q22;q22), inv(16) (p13.1q22), t(9;11) (p21.3;q23.3), t(15;17) (q22;q12), t(9;11) (p22;q23), or gene-specific somatic mutations in Fms-like tyrosine kinase (*FLT3*), *IDH1/2, P53, CEBPA, NPM1, DNMT3A, c-KIT, TET2, MLL*, and others. However, the standard of care (SOC) for AML has remained unchanged for more than five decades and involves combination of cytarabine and daunorubicin for most of the patients (3). There is no further room in escalating the intensity of chemotherapy due to the associated life-threatening toxicity. Recently the therapeutic arsenal has diversified since the advent of targeted agents against FLT3, BCL-2, and HDAC. However, the optimal strategy to use these newer drugs in isolation and in combination with chemotherapeutic agents needs to be explored further to prevent relapse and resistance to treatment.

For young adult AML patients, high-dose induction chemotherapy and allogeneic hematopoietic stem cell transplantation (Allo-HSCT) have proven to be an effective cure for around 60% of patients, but chronic toxicities and eventual relapse are common (4). The treatment options are limited for patients with refractory or relapsed (R/R) AML. The advent of risk stratification and targeted therapies in AML was possible because of extensive efforts in understanding the genetic and molecular landscape of AML. This, in turn has led to the development of new generation of drugs. However, there continue to be lack of predictive biomarkers to assign patients to suitable treatment arms (5, 6).

The concept of precision oncology stemmed from the real-world successes of imatinib in patients with BCR-ABL fusion and hormonal therapy for estrogen-positive breast cancer patients. The genomic precision medicine (GPM) approach utilizes presence of specific genetic alternations or mutations to guide treatment decisions. Although GPM has shown clinical success with EGFR or ALK inhibitors in lung adenocarcinoma, BRAF inhibitors in melanoma, and c-KIT in gastrointestinal stromal tumors, the clinical benefit in a large cohort of patients still remains obscure. Emerging data from genomics guided clinical trials such as NCI-MATCH trial, SHIVA trial, SIGNATURE program basket trial has met disappointment due to either limited assignment of patients to treatment arm or due to lack of targetable mutations or due to lower benefit (17% to 7%) in those that were assigned to the treatment arms compared to existing standard of care treatments (7–13). Recent data from NCI-MATCH trial showed that while 93% of tumor biopsies were successfully sequenced, treatment match rate was less than 26% (10). In some cases where patients were successfully matched to a treatment option, objective response rate was less than 38%, for instance in *BRAFV600E* mutated patients receiving dabrafenib and trametinib. Another randomized multicenter Phase II trial assessing the implementation of genomic precision medicine reported only 2% objective response rate (12). Ultimately, precision medicine progress so far has informed us limited clinical benefit and blunt reality that genotype alone does not reliably inform drug responses.

Over the last decade, functional precision medicine (FPM) approach has gained lot of attention due to technological advances in functional screening technologies, high-throughput platforms for drug testing, and rapid development of patient derived avatars. In FPM guided approach, the direct exposure of tumor cells to drug of choice followed by functional readout measurement is being used to rank drug sensitivity (14). This strategy does not rely on the presence of specific genetic abnormality and hence is applicable to larger patient population. While FPM approach is more generalized compared to GPM, the clinical implementation of FPM lags behind GPM. In this review, we do a narrative summary of the clinical implementation of genomic and functional precision medicine approaches for AML patients. We then delve into the future perspectives of combining both GPM and FPM strategies to further improve treatment stratification for AML.

## Genomic precision medicine approach in AML

**Figure 1:**
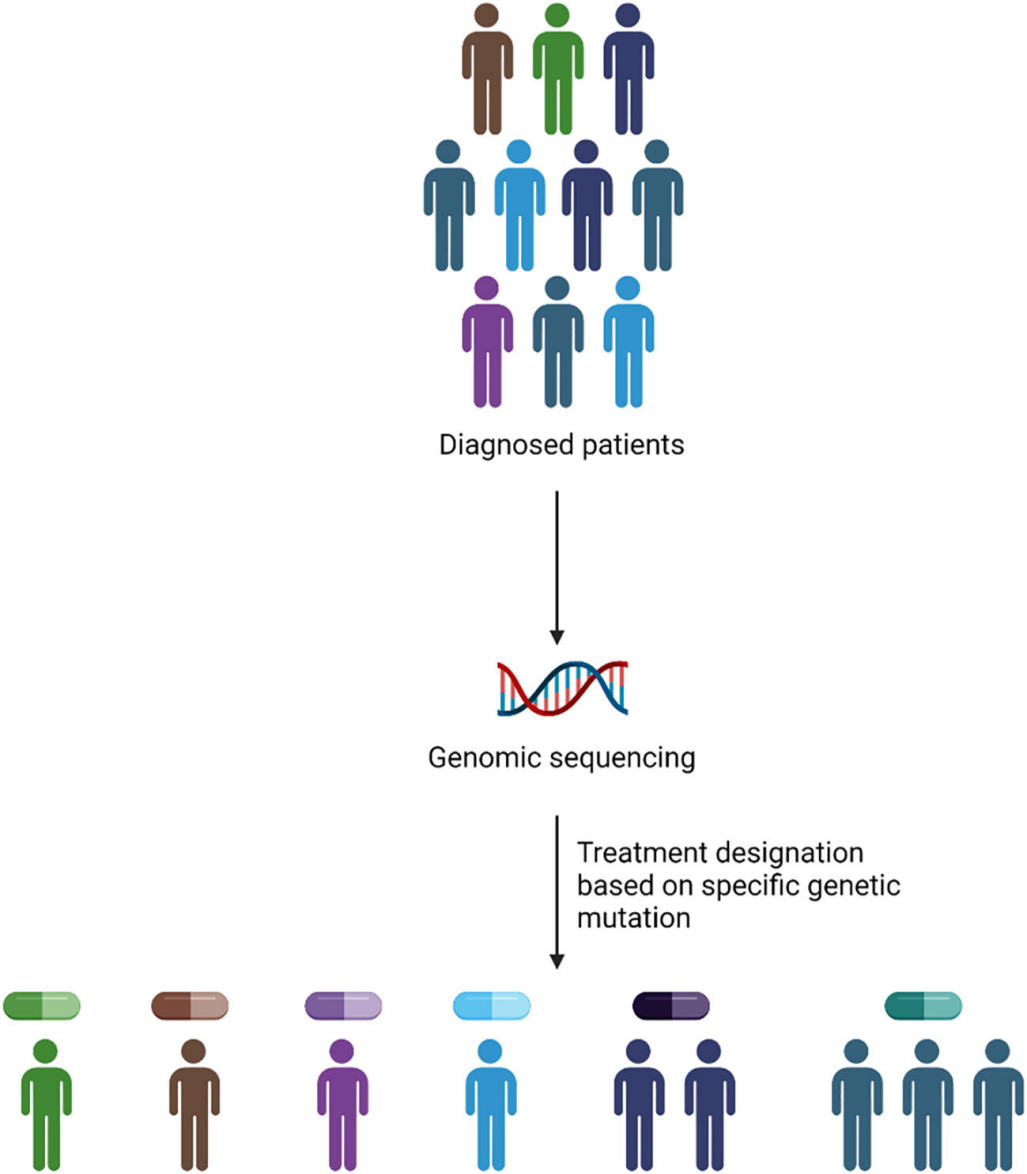
Methodology of GPM implementation in the clinic. The application of GPM depends upon the presence of genetic abnormality identified via genomic sequencing to assign personalized drug treatment.

Genomic precision medicine gained huge attention over the past two decades. With the advent of next generation sequencing (NGS), and improved RNA-sequencing, applying genomic approaches to precision medicine became cost-effective, rapid, and precise strategy to identify treatment strategies. GPM involves the identification of specific genetic mutations using NGS and assigning the right drug based on the prevalence of genetic mutation present in the patient (**Figure 1**). The identification of specific genetic marker is a key step in assigning the precise drug for the patient. Burd et al. published a landmark clinical trial (Beat AML), the first comprehensive clinical trial to leverage genomic precision medicine to assign therapy for AML patients based on the presence of somatic mutations. The assignment of different regimen based on genetic mutations showed that patients enrolled on Beat AML assigned treatment (n=224) group had significantly longer survival (median 12.8 months, 95% CI 10.3–14.8) compared to either standard of care, 7 + 3 combination (median 3.9 months, 95% CI 2.1–8.8) or palliative care (median 0.6 months, 95% CI 0.4–0.8) groups, but not significantly different from investigational therapy arm (15, 16). An expansion cohort with additional drugs including Ven-AZA is currently recruiting patients (17).

In the following section, we discuss the key genetic mutations that are currently targeted for AML treatment and the associated clinical performance.

### Targeting FLT3 mutations

About one-third of newly diagnosed AML patients carry genetic alterations in *FLT3* gene (18). *FLT3* mutations are classified into two groups: *FLT3*-ITD (internal tandem duplication), most frequent (10-25%), comprising duplication of nucleotide sequences with varying lengths and insertion sites, and *FLT3*-TKD found in 5-10% patients, comprising single-nucleotide variants in the tyrosine kinase domain (TKD) (3). Mutations in *FLT3* gene leads to constitutive activation of FLT3 signaling, causing AML cell survival, proliferation, and differentiation by regulating pathways linked to PI3K/AKT/mTOR, STAT5, and RAS/MAPK (19). AML patients carrying FLT3 mutations have inferior prognosis and are at adverse risk but outcomes vary depending on the insertion site, allelic ratio and the mutational background (20). A favorable prognosis is observed in patients with *FLT3*-TKD when it is accompanied by co-mutations in *NPM1* or core binding factor (CBF) AML patients (21). The discovery of second generation inhibitors against *FLT3*-ITD and *FLT3*-TKD has made it possible to target these mutated tyrosine kinases in AML patients.

Two classes of FLT3 inhibitors depending upon the binding conformation of FLT3 (active or inactive) are defined. RATIFY trial led to FDA approval of midostaurin, a type I inhibitor in combination with induction therapy in which the patients on combination arm showed significant improvement in event-free survival (EFS) (hazard ratio = 0.78; *P* = 0.002) and overall survival (OS) (hazard ratio = 0.78; *P* = 0.009) compared with induction therapy alone (22). Even though the RATIFY trial included only patients with age less than 60 years, the use of midostaurin is not limited to only this group of patients. The combination of midostaurin with induction or consolidation chemotherapy followed by 1-year maintenance was accessed for the efficacy in a phase III trial in *FLT3*-mutant AML adult patients (23). The OS and EFS were modestly improved with midostaurin (4-year OS 51% versus 44% for placebo), but approximately half of the patients did not benefit from the treatment due to which it has not been approved as a maintenance therapy.

Gilteritinib, a novel FLT3 inhibitor received FDA approval for R/R AML (24). Gilteritinib demonstrated significantly longer survival and higher remission rates as compared to salvage chemotherapy in patients with R/R AML (median OS 9.3 months vs 5.6 months in patients receiving salvage chemotherapy; P = 0.001). Gilteritinib also improved the relapse-free survival (RFS) of AML patients with *FLT3*-ITD after allogeneic HSCT (MJ 25). Patients with detectable minimal residual disease (MRD) showed better improvement than undetectable MRD. Crenolanib another type I FLT3 antagonist, demonstrated anti-leukemic activity in R/R FLT3 mutated AML patients in two phase 2 studies. Results from the first phase 2 trial of crenolanib with 38 patients reported a CRi rate of 23% in FLT3 inhibitor-naive patients and CRi rate of 5% in those who previously received FLT3 inhibitor. The median OS difference was also enhanced between the two groups of 55 and 13 weeks, respectively (26). The second phase 2 trial with 69 patients reported CRi rate of 39% and PR rate of 11% in relapsed FLT3-mutated FLT3 inhibitor naïve patients and CRi + PR rate of 28% in patients previously treated with FLT3 inhibitors. The median OS was 33.4 weeks in relapsed FLT3-mutated AML FLT3 inhibitor naïve patients and patients with *FLT3*-ITD had the longest overall survival (34 weeks) (27). The additional type II FLT3 inhibitor Quizartinib, type 2 FLT3 inhibitor was tested in recent clinical trial (QuANTUM-First) in combination with induction therapy in newly diagnosed *FLT3-ITD+* AML patients. In comparison to placebo plus combination, quizartinib plus combination of induction therapy showed significant increase improvement in median OS in quizartinib arm (31·9 months (95% CI 21·0-not estimable) for quizartinib versus 15·1 months (13·2-26·2) for placebo plus induction therapy (28).

Although remarkable progress is made in the discovery of highly specific and potent FLT3 inhibitors, the emergence of relapse is unavoidable (29). FLT3 inhibitor resistance is mediated by inherent or acquired mechanisms. Higher expression of FLT3 ligand in bone marrow microenvironment and low variant allele frequency of: mutated FLT3 confer resistance to FLT3 inhibitors. Activation of MAPK/STAT5 signaling, rapid metabolism by liver CYP3A4 enzyme, CYP3A4 enzyme activity in bone marrow stromal cells, acquired *FLT3*-TKD secondary mutations, loss of *FLT3*-ITD, and activation of PI3K/AKT or RAS alternative signaling are some of the resistance mechanisms reported against FLT3 inhibitors (30–36).

### Targeting IDH1/2 mutations

The frequency of *IDH1/2* mutations in AML is approximately 15-20% (37, 38). *IDH1* and *IDH2* are different isoforms of iso-citrate dehydrogenase, a key enzyme in citrate metabolism catalyzing the conversion of isocitrate to α-ketoglutarate (αKg). Mutations in *IDH1/2* causes reduction of αKg to 2-hydroxyglutarate (2-HG) which acts as a competitive inhibitor of αKg-dependent enzymes, namely TET family enzymes and lysine demethylases. The prognostic value of *IDH* mutations on AML prognosis is still actively being investigated, however, *IDH1* mutations are generally associated with an inferior treatment outcome and *IDH2* with a favorable treatment outcome (39, 40).

Multiple small molecule IDH1/2 inhibitors are currently under investigation for the treatment of AML. Ivosidenib is a first-in-class, selective, orally available, small-molecule inhibitor of *IDH1*. Ivosidenib reduces oncometabolite 2-HG levelsto induce differentiation of malignant cells (41). Ivosidenib received FDA approval for R/R AML in 2018 and newly diagnosed AML in 2019. The safety and efficacy of ivosidenib was evaluated in a phase 1 dose-escalation and dose-expansion study in *IDH1*-mutated AML R/R patients. Ivosidenib showed of 30.4% CRi(95% confidence interval [CI], 22.5 to 39.3),21.6% CR (95% CI, 14.7 to 29.8), and 41.6% ORR (95% CI, 32.9 to 50.8). The median duration of responses were 8.2 months (95% CI, 5.5 to 12.0), 9.3 months (95% CI, 5.6 to 18.3), and 6.5 months (95% CI, 4.6 to 9.3), respectively (42). Enasidenib (AG-221) is a first-in-class, oral, selective small molecule inhibitor of mutant IDH2 enzymes. AG-221 is a selective antagonist of mutant IDH2 variants R140Q, R172K, and R172S. Similar to Ivosidenib, Enasidenib also reduces of serum 2-HG level to cause myeloid differentiation (43). In 2017, enasidenib received FDA approval for *IDH2* mutated R/R AML patients. The safety, pharmacokinetic and pharmacodynamic evaluation of enasidenib was evaluated in a phase 1/2, open-label study in advanced hematologic malignancy patients with *IDH2* mutation. The overall response rate among patients with R/R AML was 40.3 percent, with a median response duration of 5.8 months. Responses were linked to cellular differentiation and maturation, although there was no indication of aplasia in most cases. The median overall survival in R/R patients was 9.3 months, and for the 34 patients (19.3%) who attained CR, the OS was 19.7 months (44).

Resistance to IDH inhibitor emerges through multiple mechanisms. Leukemia stemness plays a critical role in the primary resistance acquired to IDH inhibitors (45). Using LSC17 score Wang et al. observed a significantly higher LSC17 score in non- responders compared to responders (45). Other key mechanisms of primary resistance include epigenetic regulation, receptor tyrosine kinase (RTK) pathway mutation and MAPK pathway activation (46). The acquired resistance mechanisms to IDH1/2 inhibitors include mutations in RTK pathway, acquisition of secondary IDH mutations, isoform switching, adaptations in mitochondrial metabolism and clonal evolution/selection of resistant clones relying on other cell survival pathways (47).

### Targeting TP53 mutations

The prevalence of *TP53* mutation in *de novo* AML is around 5–10%. Chromosomal aneuploidies such as monosomy 5/deletion 5q (–5/5q–), –7/7q–, –17/17p–, complex and/or monosomal karyotypes and high-level DNA amplification are associated with *TP53*-mutant AML (48). *TP53* mutant AML patients show poor prognosis and shorter response to induction therapy. *TP53* mutations are commonly protein-altering missense mutations that exert a dominant-negative effect on p53 function. Targeting loss-of-function mutant p53 by small molecules to restore normal function of mutant p53 is adapted as therapeutic strategy (49). Eprenetapopt (APR-246/PRIMA-1Met) acts via covalent binding to specific cysteine residues (Cys124 and Cys277) in the protein core domain of mutant p53 to reactivate wild-type function of p53 (50) (51). Eprenetapopt was tested in combination with azacytidine in two separate phase Ib/II trials (n=11, and 18 respectively) in *TP53*-mutated MDS and AML patients. The trials reported CR/CRi rates of 36–45% in patients with 20–30% blasts (oligoblastic AML) and CR/CRi rate of 14% in AML patients with more than 30% blasts (52, 53). Contrary to these results, phase 3 trial showed no added benefit after combining eprenetapopt with azacitidine compared to azacitidine alone (CR rate 22.4% versus 33.3%) (54). A distinct approach of CD47 blockade via magrolimab in combination with azacytidine showed favorable outcomes in both *TP53*-mutant (40% CR, median OS 16.3 months) and wild-type patients with high-risk MDS (31% CR, median OS NR) (55). The ORR in AML patients was 71% (15/21) with 67% (14/21) of patients achieving CR/CRi. Despite initial promise, a recent randomized phase 3 (ENHANCE-2) trial, the combination of magrolimab plus azacitidine failed to show clinical benefit over venetoclax plus azacitidine or 7 + 3 chemotherapy in untreated *TP53*- mutant AML (NCT04778397).

### Targeting epigenetic enzymes

Epigenetic regulome consists of a multitude set of ‘reader’, ‘writer’, and ‘eraser’ enzymes, that results in modifications such as methylation or acetylation of histones and methylation of DNA to module gene expression in irreversible manner. DNA methyl transferase 3 *(DNMT3A)* mutations occur in ~ 20% of AML patients, making it a potential target in this subset of AML patients. In a multicentre, randomized, open label phase III trial, azacitidine (DNMT3A inhibitor) showed improvement in MOS (10.4 months with 95% confidence interval [CI], 8.0-12.7 months) when compared with standard induction therapy (6.5 months with 95% CI, 5.0-8.6 months) (56). A phase III trial compared azacytidine in combination with gilteritinib versus azacytidine alone in newly diagnosed FLT3 mutated AML patients unfit for standard of care (57). There was no significant improvement in MOS in combination arm (9.82 months) in comparison to azacytidine alone (8.87 months) but CRc was improved (4.53 in combination vs 0.03 months in azacytidine alone). Similarly, IDH1 inhibitor ivosidenib in combination with azacytidine versus azacytidine alone was tested in newly diagnosed IDH1 mutant AML patients (58). A higher EFS and MOS in combination arm than azacytidine plus placebo was observed. A comparison of decitabine vs supportive care or low-dose cytarabine in a phase III trial revealed a non-significant increase in OS but an increment in CR/CRp of 17.8% with decitabine vs 7.8% in cytarabine or supportive care (59). Based on the results of an international phase III trial (QUAZAR AML-001 Maintenance Trial) of an orally administered azacitidine derivative CC-486, FDA approved CC-486 as maintenance therapy for adult AML patients who achieved CR/CRi after intensive induction chemotherapy and who are unfit for HSCT (60).

Other than DNMT inhibitors, histone deacetylase inhibitors such as panobinostat, pracinostat, and vorinostat and lysine demethylases inhibitors such as GSK2879552 have been evaluated in AML, but the results seem disappointing when they are combined with azacitidine (61–63). Other therapeutic options include pinometostat (DOT1L inhibitor) and FT-1101 (BRD4/BET inhibitor) (64, 65). One other potential target in epigenetic therapy is lysine specific demethylase 1 (LSD1/KDM1A). The inhibition of LSD1 has been possible by an inhibitor called Iadademstat which blocks the demethylation and scaffolding function of LSD1. Inhibition of both of these functions reduces leukemic stem cell regeneration and proliferation leading to the induction of differentiation in leukemic (66, 67). Iadademstat has been tested in combination with azacidine in a phase 2 trial in AML patients (68). The investigators reported 81% of the enrolled patients responded out of which 52% had CR/CRi and 82% of CR/CRi patients evaluated for MRD were negative by flow cytometry. We have summarized the key studies and the outcomes below in **Table 1**.

**Table 1:** Summary of key studies with genomic precision medicine approach.

Regimen (e.g Chemo + drug)	Demographics	CR rate	EFS	OS	Reference
Azacitidine vs Standard Induction Therapy	Newly diagnosed AML patients (>65 years)	N/A	N/A	10.4 months vs 6.5 months in standard induction therapy	(56)
Decitabine vs supportive care or low dose cytarabine	AML patients (> 65 years)	CR/crp of 17.8% vs 7.%	N/A	No improvement in OS	(59)
CC-486	AML patients who achieved CR/CRi after intensive induction chemotherapy (≥ 55 years)	N/A	N/A	N/A	(60)
Panobinostat, pracinostat, vorinostat	R/R AML (21-71 years)	N/A	N/A	No added benefit when combined with azacitidine	(61–63)
Iadademstat + azacitidine	*De novo*/secondary AML (70-83 years)	52%	N/A	9.3 months	(68)
GSK2879552	AML patients	N/A	N/A	Disappointing results when combined with azacitidine	(61–63)
Eprenetapopt + azacitidine	*TP53*-mutated MDS and AML patients (≥ 18 years)	36-45% of patients with 20-30% oligoblastic AML and 14% in AML with >30% blasts	N/A	N/A	(52, 53)
Azacitidine + magrolimab	*TP53*-mutant and wild-type high-risk MDS patients	40% (*TP53*-mutant) 31% (wild-type)	N/A	16.3 months (*TP53*-mutant) NR (wild-type)	(55)
Magrolimab + azacitidine	*TP53*-mutant AML patients	71% (ORR) 67% (CR/CRi)	N/A	N/A	NCT04778397
Ivosidenib	Patients with relapsed of refractory AML (≥ 18 years)	30.4% (complete remission or complete remission with partial hematologic recovery)	8.2 months	NR	(42)
Enasidenib	Patients with relapsed of refractory AML (≥ 18 years)	40.3%	5.8 months	9.3 months for (R/R patients) 19.7 for patients in complete remission	(44)
Midostaurin + induction/consolidation therapy followed by 1-year maintenance	*FLT3*-mutant AML (18-59 years)	NA	Improved EFS	Improved 4-year OS of 51% vs 44% for placebo	(22)
Sunitinib + standard of care induction therapy + consolidation therapy	*FLT3*-mutant AML (≥ 60 years)	CR rate: 50% (*FLT3*-ITD) and 38% (*FLT3*-TKD)			(69)
Sorafenib monotherapy	Relapsed/refractory *FLT3-*mutated AML patients and *FLT3* wild-type patients (≤ 60 years)	10% patients achieved CR	Significant Improvement in EFS and RFS		(70)
Sorafenib + cytarabine + idarubicin	Newly diagnosed AML (18-66 years)	95% CR/Cri rates for *FLT3*-ITD positive patients	Improved DFS	Improved OS	(71)
Sorafenib + azacytidine	Relapsed/refractory AML with 93% of patiens were *FLT3*-ITD positive (24-87 years)	ORR: 46%			(72)
Gilteritinib monotherapy vs high or low-intensity salvage chemotherapy	Refractory patients (≥ 18 years)	Not specified	Not specified	Median OS 9.3 vs 5.6 months in patients receiving salvage chemotherapy	(24)
Crenolanib	Relapsed/refractory *FLT3* mutated AML patients (≥ 18 years)	23% CRi rate (*FLT3* inhibitor-naïve) 5% CRi rate (Previously received *FLT3* inhibitor)	Not specified	median OS of 55 weeks (*FLT3* inhibitor-naive) median OS of 13 weeks (previously received *FLT3* inhibitor)	(26)
Crenolanib	not specified	39% (*FLT3* inhibitor naïve) 28% (previously treated with *FLT3* inhibitors)	not specified	Median OS 33.4 weeks (*FLT3* inhibitor naïve) *FLT3*-ITD patients had the longest OS of 34 weeks	(27)
Quizartinib	(≥ 18 years)	CRc rate of 46-56%	ORR of 74-77%	Improved OS in responders compared to non-responders	(73)
Quizartinib	<60 years old with relapsed AML on first-line chemotherapy within one year	CRc rate of 56%	ORR of 77%	not specified	(73)
Quizartinib	≥18 years old with relapsed AML to second-line salvage chemotherapy or HSCT	CRc rate of 46%	ORR of 74%	not specified	(73)
Quizartinib	≥18 yearsold R/R patients	CRc rate of 47%	not specified	median OS 6.2 months vs 4.7 months in chemotherapy group	(74, 75)

N/A, Not available.

## Challenges associated with GPM approach

### Limited response rate

While GPM implementation led to a number of treatment successes in AML, only a small fraction of patients shows durable and deeper remissions from GPM assigned therapy. In *FLT3* mutated patients, FLT3 inhibitors show overall response rate of ~ 49% (76). Despite promising clinical efficacy of FLT3 inhibitors, overall survival (OS) in *FLT3*-mutated AML vs WT is similar (77). Despite existing efforts to identify new mutations and development of targeted therapies against new targets, treatment-resistant cancers continue to emerge where non-genetic factors may also drive cancer development (78). Monotherapy targeting specific genetic abnormality is seldom effective to achieve complete response. Hence combination therapy against multiple target is generally preferred (79, 80). However, genomic sequencing alone often fails to assign combination therapy due to limited number of targetable mutations or lack of drug targeting novel genetic abnormalities.

Apart from the limited clinical benefit of genomic precision medicine, we also considered reasons that hinders the implementation of genomic precision medicine in the clinical setting. McCarthy et al. highlighted the issue of clinical validity pertaining to the use of genomic precision medicine (78). Here, clinical validity was referred as how consistent and accurate genomic testing in the clinical settings. The discovery and validation of biomarkers for each cancer type is an arduous process that requires large number of patients. The issue of identifying robust biomarker can be magnified in rare cancers with fewer number of enrolled patients (78). Another challenge with clinical implementation of GPM approach is physician’s lack of knowledge in the field of genomic precision medicine (81, 82) and even if they are well versed in the field of genomic precision medicine, there may still be uncertainties when interpreting genomic data (83–85) and choosing the most appropriate treatment. Due to the heterogeneous nature of a tumor, it can be challenging to characterize the genetic mutations in the tumor (86, 87) and to identify driver mutations that do not contribute to cancer growth (78). Interpreting these genomic data may not be easy and assigning treatment may be an issue. Whilst we appreciate that the concerns of clinical validity, lack of biomarkers have been researched in solid tumors; the concerns also apply to hematological malignancies in our opinion. Cumulatively, these factors hinder the clinical implementation of genomic precision medicine.

### Co-mutations and non-targetable genetic changes

GPM success from the assigned targeted therapies relies on the presence of specific mutations. However, individual prognostic information derived from the presence or absence of a specific mutation does not take into account cooperating co-mutations that may cause altered oncogenic dependence and signaling (88). In a recent mutational spectrum study, over 90% of *NRAS* mutant AML patients had at least one co-mutation. Furthermore, treatment of patients with recurrent genetic mutations including *FLT3*-ITD, *NPM1*, *DNMT3A*, *TET2* and *KIT* and fusion genes including *AML1-ETO* and *MYH11-CBFβ* are found to be unfavorable due to possible interplay between mutated genes (89). Adverse outcomes were observed in patients with *DNMT3A* and *FLT3*-ITD co-mutations (90). Beyond co-mutations, mutational burden of individual genes (VAF), can often play a key role in dictating responses to targeted therapy. Studies on the impact of clonal burden of *TP53*, *TET2*, *DNMT3A*, and *NPM1* show that high VAF is associated with a shorter survival time (91). Absence of good predictive markers as well as the presence of non-targetable co-mutations are posing a big challenge to current GPM approaches.

### Time to assignment of therapy

While recent technological advances has reduced the turnaround time for NGS data, it still requires several days to few weeks for genomic screening results. As opposed to indolent tumors, AML is a highly aggressive malignancy and requires immediate initiation of therapy to minimize disease-related morbidity and mortality (92). Data from a recent study that compared time from diagnosis to treatment showed no significant difference in OS based on treatment time delays (93). Hence it is still a debatable whether increase in time to treatment after diagnosis until sequencing data is obtained to enroll patients on GPM based treatment is safer option and will not affect assigned treatment outcomes. That being said having more rapid method of screening for AML-specific genetic mutations is desirable for rapid deployment of targeted therapies. Another caveat is that genomic sequencing provides a snapshot of a static event in the cell. Myeloblasts in AML bone marrow are dynamic, consisting of microenvironment as well as soluble factors. Due to this, the drug predicted by GPM often fail produce desired therapeutic effect despite of presence of genetic mutation. Often polyclonal heterogenous tumors upon relapse become oligoclonal with a complex genomic phenotype (94). Assignment of the precise drug to eradicate this complex population is a daunting task by genomic approach because of multiple co-genetic mutations, thus a combination of drugs, identified by testing multiple drugs using alternative precision medicine approaches are desired.

## Functional precision medicine approach

FPM is underpinned by the idea that biological systems are exceptionally complex and predicting cellular response with initial conditions of genetic mutations can often be challenging and inaccurate. The core driving principle of functional precision medicine (FPM) relies on the perturbation of a system followed by the measurement of generated response (95).

**Figure 2:**
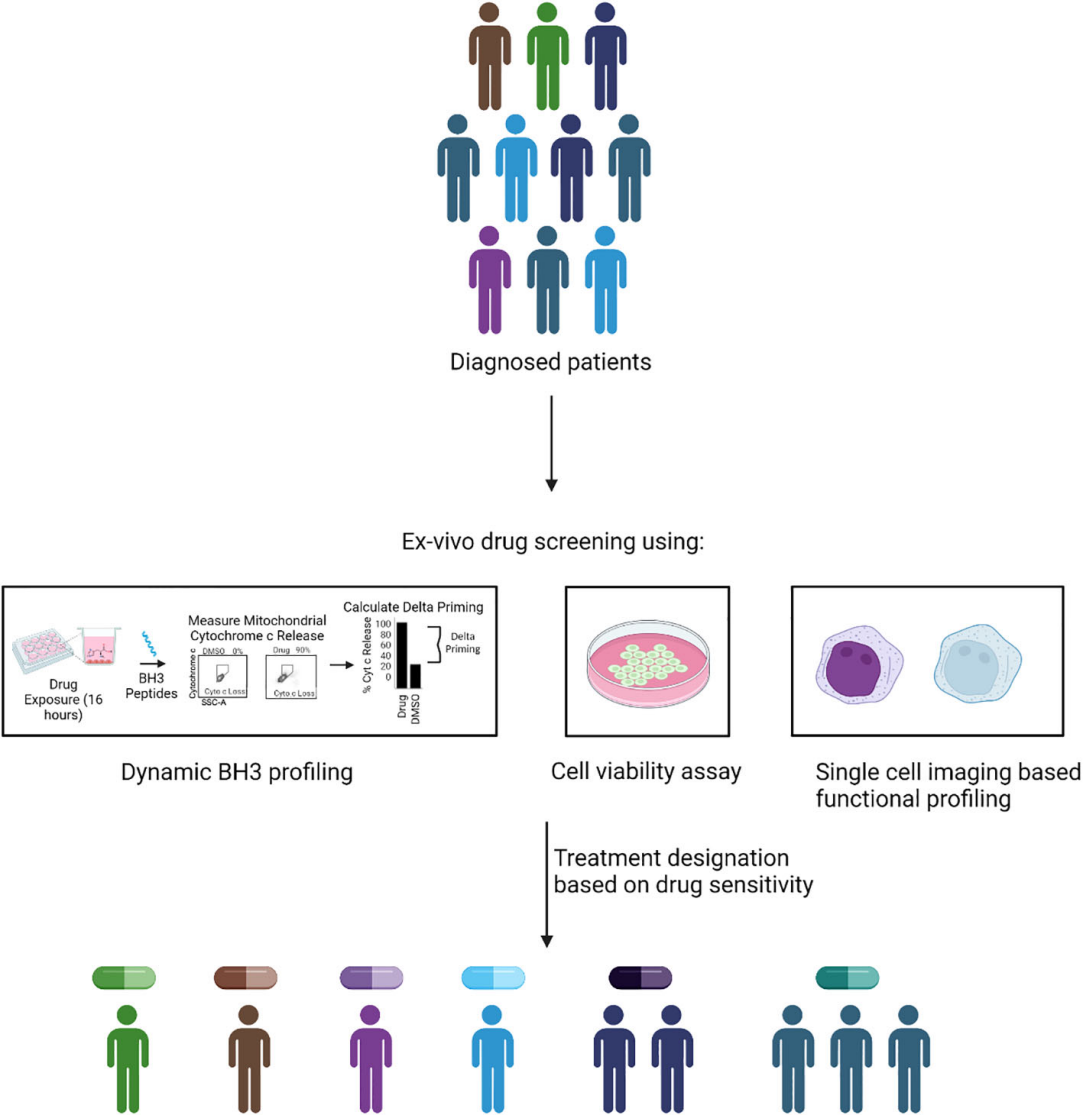
Methodology of FPM. In FPM approach, drug treatment is assigned based on the ex-vivo functional screening of isolated tumor cells. The functional screening could be done via different methods such as dynamic BH3 profiling, cell viability assay or imaging based functional phenotype assessment. The quantifiable read of the assay such as increase in apoptotic priming, reduced cell viability and functional change in phenotype is used assign treatment for patients based on their individual response.

FPM aims to revolutionize concept of precision medicine in which rather than relying on genetic biomarkers and mutations, it directly leverages tumor samples from patients to assess sensitivity to a multitude of anti-cancer agents. A functional measurement-based precision medicine provides particular advantage for cancers with poor response to drugs and high inter and intra-tumor heterogeneity, such as AML. Currently, FPM approach is implemented by using multiple approaches to identify personalized cancer therapy (**Figure 2**). In the subsequent session we discuss various FPM methods.

## Methods for FPM

### Assigning BH3 mimetics therapy using BH3 profiling

Although mutations in BCL-2 was not identified as a putative genetic marker to assign therapy to AML patients, targeting BCL-2 has become one of the most successful targeted therapy in AML in current clinical practice. While evasion of apoptosis was documented as one of the hallmarks of cancer (96), its relevance in therapeutic targeting was not realized until implementation of FPM approach called BH3 profiling. BH3 profiling measures mitochondrial priming of cancer cells to determine threshold to apoptosis. Using BH3 peptides specific to proapoptotic BH3 only proteins, BH3 profiling can also infer anti-apoptotic dependence of specific cell type (97, 98). The cellular fate of survival or apoptosis is based on the balance between the BCL-2 family pro-survival proteins and the proapoptotic BH3-only proteins. Under the activation of proapoptotic BH3-only proteins, BAX and BAK oligomerizes on mitochondrial outer membrane to form pores followed by release of cytochrome c and downstream activation of caspases (99). The discovery of *BCL-2* as oncogene which had anti-apoptotic activities, its binding with BH3 only pro-apoptotic proteins, and further reports of evidence showing the selection of malignant cells with high BCL-2 expression has led to the stimulus for targeting BCL2 via small molecule inhibitors such as BH3 mimetics (example venetoclax) (100, 101).

First-in-class BH3 mimetic developed to target BCL2 family protein was ABT-737 which has a broad-spectrum activity against the BCL-2, BCL-XL, BCL-XL and BCL-W. Using BH3 profiling approach, functional dependence on BCL-2 was first documented for chronic lymphocytic leukemia (CLL) which led to clinical testing of navitoclax in CLL patients (102). Despite of remarkable clinical activity, further progress was halted due to the on-target inhibition of BCL-XL that led to severe thrombocytopenia (103, 104). Further search for BH3 mimetics with BCL-2 specificity led to the discovery of highly potent BCL-2 inhibitor ABT-199 (Venetoclax) (105–108). By using BH3 profiling once again venetoclax showed on-target cytotoxicity for CLL cells at IC50 <10nM and this work led to clinical trial program that ultimately yielded multiple regulatory approval for venetoclax in CLL (102, 109). While AML cells did show evidence for functional dependence on BCL-2 using BH3 profiling, compared to CLL, AML myeloblast had heterogenous dependence (110). Clinical evaluation of venetoclax as a single agent in R/R AML patients revealed a short-lived response with a CR/CRi rate of 19% (111). The combination of venetoclax with LDAC resulted in a CR/CRi of 54% with a median time to first response of 1.4 months. The median OS was 10.1 months (95% CI, 5.7 to 14.2) (112); with HMA, the CR/CRi was 73% and 67% of all patients achieved CR (113, 114). The combination of venetoclax with IDH1 inhibitor ivosidenib in *IDH1*-mutant AML, with gilteritinib in *FLT3* mutant AML patients, and with a MEK inhibitor has also been explored by (115–118). Stevens et al., have shown that the emergence of resistance against venetoclax and azacitidine combination is due to the upregulation of fatty acid metabolism via RAS pathway mutations or compensatory adaptations (119). The monocytic resistant clones lost the BCL-2 expression and were relying on MCL-1 for survival (120). Genomic deletion of BAX leading to venetoclax resistant MV4-11 AML cell lines have also been reported (121) as a resistance mechanism. Additional mechanisms of resistance by reduction in overall apoptotic priming in venetoclax resistant PDX cells and adaptive apoptotic rewiring via sequestration of pro-apoptotic proteins by MCL-1/BCL-XL rather than BCL-2 and/or decreased BAX expression have also been reported (122). Other than BCL-2, inhibitors targeting other specific BCL-2 family proteins like MCL-1, and BCL-XL are also being explored. (123–127).

### Dynamic BH3 profiling and BH3 profiling assay

DBP method measures drug-induced apoptotic priming (delta priming) after treatment with proapoptotic BH3 peptides (128). In this approach, isolated cancer cells or tumor tissues are first exposed to drugs for 8-24 hours, followed by cell membrane permeabilization with digitonin and subsequent exposure to BH3 peptides derived for proapoptotic BH3 domains. Drug response is expressed as difference in cytochrome c release to indicate mitochondrial outer membrane permeabilization. The utility of DBP to accurately predict functional response of the cells has not been limited to hematological malignancies but was also implemented in solid tumors (129–132).

DBP approach was applied to discriminate the responders from non-responders in lenalidomide (LEN) plus MEC (mitoxantrone, etoposide, and cytarabine) combination chemotherapy inR/R AML patients. Increased mitochondrial signaling measured via DBP after a short-term ex vivo exposure of LEN to pretreatment myelobalsts was able to discriminate clinical responders from non-responders (133). To overcome resistance against venetoclax and S63845, Bhatt and colleagues utilized BH3 profiling and DBP and reported that the drug resistance is attributed to the reduction in mitochondrial apoptotic priming (122). Next they applied DBP as an FPM approach to identify the personalized treatment option for resistant models by exposing the myeloblasts isolated from AML PDX models to a panel of 40. DBP revealed that FLT3 inhibitors, SMAC mimetics, HDAC inhibitors, CDK9 inhibitors and MCL-1 inhibitors could be used to overcome the venetoclax resistance (122).

### Ex vivo drug sensitivity assay

A functional screening of either single agent or combinations via measurement of cell viability has been extensively tested for efficacy in tailoring personalized medicine since decades. Using latest technologies and sophisticated culture conditions, several independent groups carried out ex vivo analysis of tumor cells to guide therapy for blood cancers (134–139). Kurtz et al. (139) applied ex vivo drug sensitivity assay to AML and CLL primary tumours and found that combinations involving kinase and MEK inhibitors were favorable in AML. They also correlated the combination ratio scores with patients’ genetics and other clinical markers and reported that certain combinations were specific to genetic markers but many combinations were effective irrespective of the genetic makeup of the patients, suggesting the relevance of FPM in identifying clinically effective combinations. Likewise, another study that utilized 151 primary leukemia samples to test panel of kinase inhibitors via *in vitro* cell viability assays, reported that functional vulnerability to kinase inhibitor may or may not be correlated to genetic abnormality (134). The Beat-AML study discussed in GPM also included ex-vivo drug sensitivity profiling and corelated the drug response to genomics and transcriptomics of the myeloblasts (15). The initial Beat-AML cohort was expanded with an expansion cohort with size to 805 patients was recently published by Bottomly and colleagues showing correlation between transcriptomics and AML cell differentiation state regulating the ex-vivo drug response (140). The same group has published a recent study evaluating 25 venetoclax inclusive combinations in AML to overcome resistance to venetoclax and azacitidine (141). They utilized ex vivo drug screening to test the combinations and correlated the drug response to tumor cell differentiation state.

Malani et al. implemented first of its kind ex vivo drug-sensitivity response (DSRT) assay to first time evaluate the clinical efficacy of functionally assigned therapy in AML patients. By setting up functional precision tumor board (FPTMB) for AML, Malani and colleagues integrated ex vivo monotherapy responses against 515 anticancer drugs with clinical, molecular, and genomic data for a total of 252 primary AML tumors collected from 186 patients (138). Based on the specific criteria devised in the study, four major drugs (venetoclax, BCL2i; sunitinib, VEGFR/FLT3i; dasatinib, BCR-ABL1/other kinases inhibitor, and temsirolimus mTORi) were tested for the clinical implementation of the FPMTB data. The drugs combinations (two-three drugs) were administered based on patient-specific sensitivity to single drugs and molecular data. Leveraging the FPMTB guided therapy, successful responses were recorded in 59% of R/R AML (n=29), of which 45% of patients (n=13) achieved CR/CRi. Of note, the DSRT assay provided actionable data in 3 days, faster than genomic and transcriptomic profiling, recommending rapid implementation of treatment options in patients who have failed the standard therapy and those requiring urgent alternative therapy options.

In another approach, the functional therapy assignment was leveraged based on drug responses were quantified via high-content microscopy at single-cell resolution in patient biopsy samples (137). A first-in-class prospective trial, Extended Analysis for Leukemia and Lymphoma Treatment (EXALT), was conducted to evaluate image-based single-cell functional precision medicine (scFPM) approach in guiding treatments in 143 patients with advanced aggressive hematologic cancers (137). scFPM approach assigned fifty-six patients (39%) for subsequent therapy and reported 54% patients (30 out of 56) achieved clinical benefit with PFS > 1.3 months after a median follow-up of 23.9 months. Encouragingly, scFPM-matched therapy resulted in a mean PFS of 276 days compared with 121 days on their previous treatment (P =0.0039), whereas nonmatched therapy led to a mean PFS of 96 days with a mean previous PFS of 121 days (*P* = 0.51).

The application of ex-vivo drug profiling or pharmacotyping has also been applied in acute lymphoblastic leukemia (ALL). Gocho et al., reported ex-vivo functional screening of 352 pediatric and adult T-ALL patients to characterize the sensitivity of dasatanib, a BCR-ABL kinase inhibitor (136). Although T-ALL patients do not show BCR-ABL fusion, the study reported that the functional screening with dasatanib revealed sensitivity in 44.4% of pediatric T-ALL patients, indicating the importance of FPM based approaches in identifying effective therapy. They further characterized dasatinib sensitivity in T-ALL patients by network-based systems pharmacology to examine the signal circuitry and reported that preTCR-LCK activation was correlated with dasatinib sensitivity. A similar approach of pharmacotyping was done in 805 pediatric ALL primary samples with 18 drugs to define whether leukemia genomics and MRD status influence drug sensitivity (142). They identified distinct genomics clusters and MRD level contributing to the drug sensitivity in B-ALL and T-ALL pediatric patients.

FPM approach has been extended to multiple myeloma (MM) to devise new combinatorial treatment. Rashid et al., reported a quadratic phenotypic optimization platform (QPOP) to identify new combinatorial treatments based on the ex-vivo screening of 114 approved drugs (135). The uniqueness of this approach lies in its methodology in which no molecular mechanisms or predetermined drug synergy data is required. This approach utilized quadratic surfaces to model the biological effects of drug combinations to identify effective drug combinations. They successfully utilized QPOP to devise new combinations in bortezomib resistant MM cell lines and xenograft mouse models.

The next major question is whether FPM is clinically feasible to guide the treatment in prospective fashion. A prospective non-interventional SMARTrial that tested the clinical feasibility and predictive powers of ex vivo drug response profiling addressed that it is indeed possible to prospectively assign therapy using FPM approach (143). SMARTrial tested end points in which the primary endpoint provided drug response in 7 days and the secondary endpoint correlated the findings of the ex-vivo response to the *in vivo* drug activity in patients. Investigators reported that the trial met the primary endpoint in 91.3% (95% confidence interval (95% CI) 82.8–96.4%) of all eligible participants. To associate the ex vivo drug sensitivity and *in vivo* response, they conducted logistic regression on ex vivo drug response profiles of individual chemotherapeutic agents to *in vivo* response and reported a median cross-validation area under the receiver operating characteristic curve (AUROC) of 0.84 to 0.85 with a model of 5 drugs used as prognostic features. They also regressed EFS to the ex vivo response and reported a stronger ex-vivo response was associated with extended EFS. They also validated this approach in 95 AML patients reporting a stronger correlation between *in vivo* response to cytarabine and daunorubicin and ex vivo drug sensitivity.

An independent clinical trial, VenEx tested the utility and predictiveness of venetoclax ex vivo sensitivity in *de novo* (patients ineligible for induction therapy), R/R or secondary AML patients (144). The study reported that the cell culture media also influences the ex vivo drug sensitivity profile and condition media based on RPMI with supernatant from stroma cells outperformed. The treatment response was achieved in 88% of the ex-vivo drug sensitive patients. They also reported a significant longer median survival for participants who were ex vivo-sensitive to venetoclax (14.6 months for venetoclax-sensitive patients vs. 3.5 for venetoclax-insensitive patients, P<0.001). However, ability of VenEx study to prospectively identify insensitive patients remained to be determined via ROC analysis (144).

Assigning treatment based on the mutational status of the patient is not productive as the underlying biology of the tumor is extensively complex. Therefore, a combination of genetic and functional predictive biomarkers is needed to guide the therapy decision. Some of the predictive biomarkers for specific agents are provided in **Table 2**.

**Table 2:** Predictive biomarkers reported for therapy selection of targeted agents.

Drug	Biomarkers for sensitivity	Biomarkers for resistance	Reference
Venetoclax	*BCL2, PML-RARA*, *WT1*, *FLT3*+*IDH1*	*CLEC7A* (CD369), CD14, *KRAS*, *TET2, SF3B1, PTPN11*, and *BCL2A1*	(145)
	*NPM1, IDH1/2* and R/R *RUNX1*	Monocytic phenotype, pre-treatment with hypomethylating agents, BCL-2 mutation, MCL-1 dependency, TP53 mutation with complex karyotype	(146)
FLT3-ITD inhibitors	*NPM1, DNMT3A*		(147)
JQ1 (BET inhibitor)	*FLT3-ITD/TKD*	*IDH1/2, TET2*, and *WT1*	(148)
MDM2 inhibitor	miR-10a		(149)

## Challenges associated with FPM approach

FPM approach provides significant advantage over GPM by excluding the inherent requirement of presence of genetic abnormality. FPM enables generalized applicability of the approach to broader population of patients. But there remain inherent challenges to FPM approach that needs to be overcome in the coming years. Firstly there is the requirement for viable tissues. For multicentre prospective clinical studies, it is imperative to optimize the storage as well as transport conditions to ensure good tissue viability. Also, since viable tissues are required, there needs to be enough cells for implementation of FPM (95). It is also difficult to optimize drug concentration and treatment duration and interval to be used for ex vivo FPM testing as the drug concentration varies across patients due to interindividual differences in pharmacokinetics (metabolism and elimination) (95). Since ex-vivo testing is done away from the tumor microenvironment, there remains a possibility that the predicted drug combinations maybe less efficacious in patients. Therefore, model used for FPM testing must be carefully considered. In case of organoid models, tumor microenvironment cells are excluded. These cells play a role in regulating drug responses (150) and therefore, this method does not truly represent the tumor and the interaction with their tumor microenvironment. PDX models may exhibit a more realistic tumor microenvironment, however, human tumor cell-stroma cell interactions are affected with the original human stromal cells gradually replaced by murine stromal cells (151). Importantly interaction with immune cells cannot be tested in PDX models as these models are derived in immunodeficient mice (152). FPM testing may take longer time in certain approaches, especially if ex vivo cell expansion is required. To truly adopt FPM as a global standard, better standardization practices pertaining to doses, time to measure perturbance, tumor mass need to be established for it to produce reliable and replicable results need to be addressed rigorously.

## Looking beyond genomic and functional approach

GPM and FPM have made an impact in targeting complex tumor biology, but both approaches have inherent limitations. Because of rapid development in technology, a precision medicine approach based on omics technology (proteomics or transcriptomics) might become a possibility in future years. As deregulation of different types of proteins such as signaling or sensor proteins is usually observed at the transcriptional (in terms of RNA transcripts) or translational level in disease state, identification and targeting these specific set of vulnerabilities is a possible way of delivering precision medicine for the patients. Proteomics based precision medicine would require mass spectrometry or high throughput affinity-based methods like ELISA, aptamers or antibody labelled nucleotide arrays to quantify the proteins in individual patient samples (153). Integration of genomic with transcriptomics and proteomic data will be a better strategy to understand the significance of genomic alterations at the translational level and to better identify biomarkers for effective targeting (154). Beyond this state-of-the-art single cell RNA sequencing (scRNA-seq) may dwell upon in near future to aid personalized medicine. Single cell technology will offer a unique advantage of deconvoluting clonal heterogeneity of the bulk tumor to specific subset of tumor population. Such knowledge could enable identification of unique biomarkers in different clusters of tumor population to deliver precision medicine to target clonal heterogeneity and thus the emergence of resistance clones. A detailed description of transcriptomics based precision medicine has been reviewed elsewhere (155, 156).

## Future perspectives

The evolution of different treatment strategies allude to the ever-growing nature of precision medicine. As our understanding of AML improves, so would the strategies undertaken to address it. Although a genetic approach to precision medicine has improved clinical outcomes for specific subgroup of patients carrying mutations, its ability to assist in guiding therapy for a broad spectrum of patients remains limited. This limitation becomes especially evident when used in a heterogeneous cancer like AML. In comparison, functional precision medicine allows identification of personalized regimens that do not rely on mutations or the genomic makeup of a patient, allowing for much broader implementation. However, functional approaches also have limitations that need to be addressed in coming years. These approaches require fresh tumor samples and are still in infancy due to requirement for robust standardization. However, in the coming years, field will improve on the shortcomings and will improve on the existing functional approaches to enable broader use of it as predictive biomarker.

**Figure 3:**
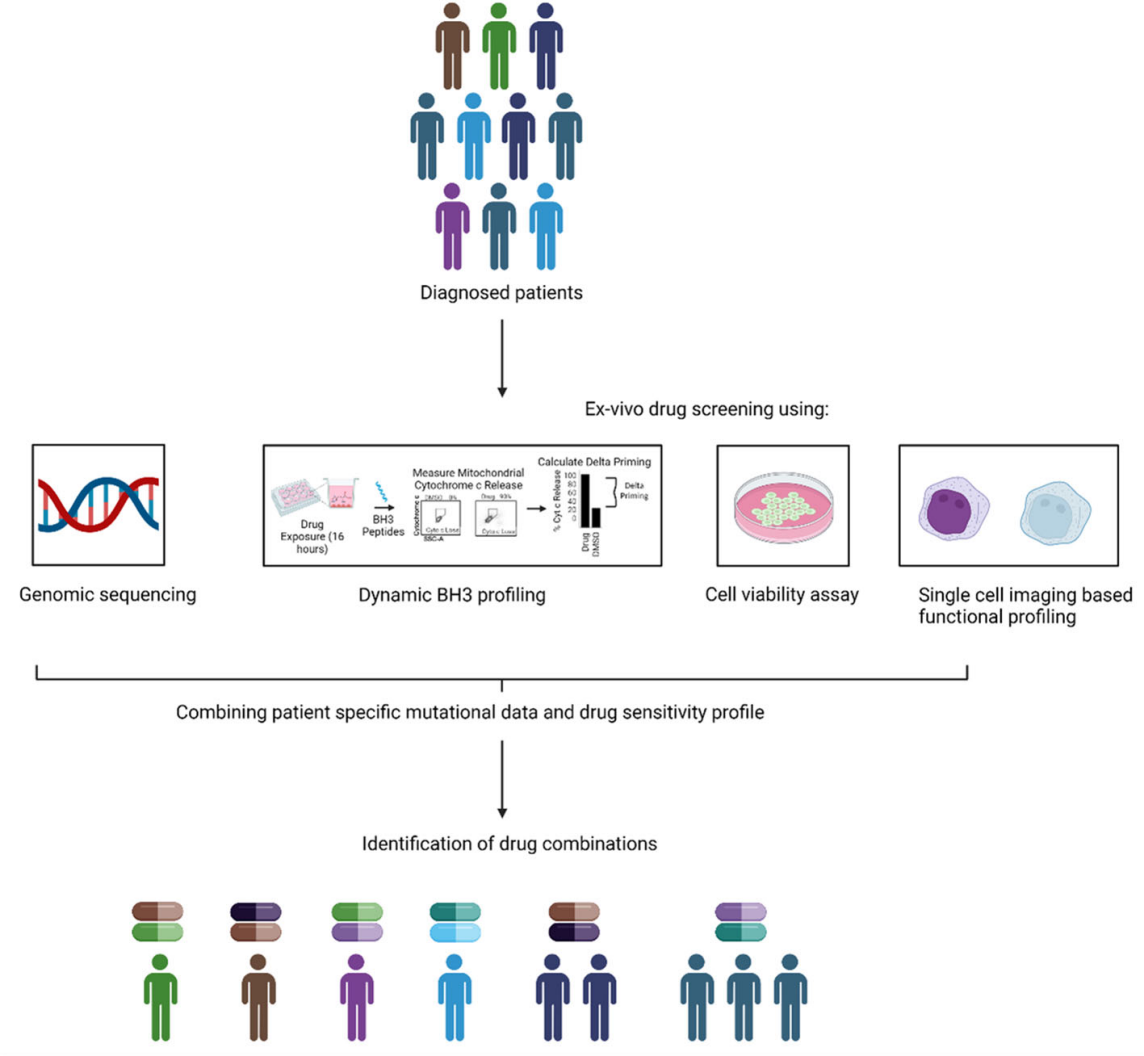
Combining genomic and functional precision medicine. An alternative to the current GPM and FPM based approach is to combine them together to assign therapy. In the combination approach, the genomic and functional data could be combined to guide the treatment to design new and precise combinations of the drugs for individual patients.

Integrating functional approaches with genomics would not only yield greater insight into the biology of AML, as observed by Malani et al. but also allow for better treatments to be designed for AML. We posit a growing convergence of these two techniques in clinical trials over the coming years (**Figure 3**). This would allow the use of FPM and GPM, working in tandem to potentially confer a greater degree of benefit to patients with AML. A recent example of such case is the use of triplet therapy consisting of venetoclax, hypomethylating agent (decitabine) and *FLT-3* inhibitor gilteritinib in *FLT-3* mutated AML. A phase-II trial validated this combination in newly diagnosed and relapsed/refractory patients and reported the CRc rate of 92% with MRD negativity by FCM in 56% and by PCR/NGS in 91% of responders in newly diagnosed group and the CRc rate of 62% with MRD negativity rate by FCM in 63% and by PCR/NGS in 100% of responders in R/R group (157). The enormous data generated through such precision medicine approaches could help to identify and develop novel drug combinations using machine learning (ML) algorithms. ML could help in the identification of synergism between drugs and also help to predict if a specific genetic signature is correlated with a selected drug sensitivity profile to a panel of drugs. One example of such study was reported by Lee and the colleagues., in which they integrated *in vitro* drug sensitivity to 160 chemotherapy drugs in 14 AML cell lines and genome wide-gene expression profiles of 30 AML patients to identify molecular markers in guiding the treatment of AML patients (158). Their machine learning approach was named MERGE based on its utilization of mutation, expression hubs, known regulators, genomic CNV, and methylation data to predict the gene-drug association. They compared MERGE with existing conventional methods and compared the consistency rate (number of significant gene-drug associations predicted by each method) and reported a higher consistency rate with MERGE. They also utilized MERGE to predict the molecular markers to topoisomerase II inhibitors and reported *SMARCA4* as a potential marker in AML driving the sensitivity towards topoisomerase II inhibitors. Other such studies predicting the sensitivity of a drug combination and overall application of ML has been reviewed here (159).

The success of GPM, FPM or combination based therapy would also depend on the diversity of population enrolled in clinical trials. Increasing representation of minority and underserved populations in clinical trials is important to overcome inherent bias in such studies. To improve representation, policy makers, healthcare providers and insurance providers need to work in tandem.

Extraordinary progress has been made in the understanding of predictive biomarkers as well as AML biology. This, in combination with recent approaches in integrating synergistic therapies with novel agents and the use of ML algorithms promises a successful future in improving outcomes in AML using personalized medicine approaches.

## Author contributions

KB: Conceptualization, Writing – original draft, Writing – review & editing. VS: Writing – original draft. MW: Writing – original draft. PI: Conceptualization, Supervision, Writing – original draft, Writing – review & editing. SB: Conceptualization, Supervision, Writing – original draft, Writing – review & editing.
